# Synergism of peptide receptor-targeted Auger electron radiation therapy with anti-angiogenic compounds in a mouse model of neuroendocrine tumors

**DOI:** 10.1186/2191-219X-4-9

**Published:** 2014-02-16

**Authors:** Andreas Wicki, Damian Wild, Vincent Prêtre, Rosalba Mansi, Annette Orleth, Jean-Claude Reubi, Christoph Rochlitz, Christoph Mamot, Helmut R Mäcke, Gerhard Christofori

**Affiliations:** 1 Department of Medical Oncology, University Hospital Basel, Petersgraben 4, Basel CH-4031, Switzerland; 2 Department of Biomedicine, University of Basel, Petersgraben 4, Basel CH-4031, Switzerland; 3 Department of Radiology, Division of Nuclear Medicine, Basel University Hospital, Basel CH-4031, Switzerland; 4 Division of Radiological Chemistry, Basel University Hospital, Basel CH-4031, Switzerland; 5 Institute of Pathology, Berne University Hospital, Berne CH-3010, Switzerland; 6 Division of Oncology, Cantonal Hospital, Aarau CH-5001, Switzerland; 7 Department of Biomedicine, Institute of Biochemistry and Genetics, University of Basel, Basel 4031, Switzerland

**Keywords:** Glucagon-like peptide receptor, Neuroendocrine tumors, Anti-angiogenesis, Auger emitter

## Abstract

**Background:**

Neuroendocrine tumors are well vascularized and express specific cell surface markers, such as somatostatin receptors and the glucagon-like peptide-1 receptor (GLP-1R). Using the Rip1Tag2 transgenic mouse model of pancreatic neuroendocrine tumors (pNET), we have investigated the potential benefit of a combination of anti-angiogenic treatment with targeted internal radiotherapy.

**Methods:**

[Lys^40^(Ahx-DTPA-^111^In)NH_2_]-exendin-4, a radiopeptide that selectively binds to GLP-1R expressed on insulinoma and other neuroendocrine tumor cells, was co-administered with oral vatalanib (an inhibitor of vascular endothelial growth factor receptors (VEGFR)) or imatinib (a c-kit/PDGFR inhibitor). The control groups included single-agent kinase inhibitor treatments and [Lys^40^(Ahx-DTPA-^nat^In)NH_2_]-exendin-4 monotherapy. For biodistribution, Rip1Tag2 mice were pre-treated with oral vatalanib or imatinib for 0, 3, 5, or 7 days at a dose of 100 mg/kg. Subsequently, [Lys^40^(Ahx-DTPA-^111^In)NH_2_]-exendin-4 was administered i.v., and the biodistribution was assessed after 4 h. For therapy, the mice were injected with 1.1 MBq [Lys^40^(Ahx-DTPA-^111^In)NH_2_]-exendin-4 and treated with vatalanib or imatinib 100 mg/kg orally for another 7 days. Tumor volume, tumor cell apoptosis and proliferation, and microvessel density were quantified.

**Results:**

Combination of [Lys^40^(Ahx-DTPA-^111^In)NH_2_]-exendin-4 and vatalanib was significantly more effective than single treatments (*p* < 0.05) and reduced the tumor volume by 97% in the absence of organ damage. The pre-treatment of mice with vatalanib led to a reduction in the tumor uptake of [Lys^40^(Ahx-DTPA-^111^In)NH_2_]-exendin-4, indicating that concomitant administration of vatalanib and the radiopeptide was the best approach. Imatinib did not show a synergistic effect with [Lys^40^(Ahx-DTPA-^111^In)NH_2_]-exendin-4.

**Conclusion:**

The combination of 1.1 MBq of [Lys^40^(Ahx-DTPA-^111^In)NH_2_]-exendin-4 with 100 mg/kg vatalanib had the same effect on a neuroendocrine tumor as the injection of 28 MBq of the radiopeptide alone but without any apparent side effects, such as radiation damage of the kidneys.

## Background

In neuroendocrine tumors, a range of different cell surface markers is expressed preferentially [1,2]. The best-known example is the somatostatin receptor subtype 2 (sst_2_), which is expressed on most neuroendocrine tumors and can selectively be targeted by the somatostatin analogue octreotide. Coupling of octreotide derivatives to a radionuclide selectively induces cell death in somatostatin receptor-expressing tumor cells [3,4]. As a new target, the glucagon-like peptide-1 receptor (GLP-1R) has emerged. GLP-1R is highly expressed on insulinomas, gastrinomas, phaeochromocytomas, and other neuroendocrine tumors [1,5], and [Lys^40^(Ahx-DTPA-^111^In)NH_2_]-exendin-4, a radiolabelled GLP-1 analogue, is specifically internalized in GLP-1R-expressing tumor cells [6,7]. [Lys^40^(Ahx-DTPA-^111^In)NH_2_]-exendin-4 is a radiopharmaceutical that consists of exendin-4 and the chelator diethylene triamine pentaacetic acid (DTPA), which in turn binds to ^111^indium, a γ-emitter and Auger emitter. The dense shower of short-range Auger electrons (range below 0.5 μm) released by ^111^In results in biological damage that is highly dependent on the location of the decay site within the cell. Optimal Auger radiation efficacy is obtained when Auger emitters are tightly bound to DNA.

We have shown in the Rip1Tag2 mouse model of pancreatic neuroendocrine tumors (pNET) that [Lys^40^(Ahx-DTPA-^111^In)NH_2_]-exendin-4 is suitable for molecular imaging of NETs using single-photon emission computed tomography (SPECT). pNETs could be detected down to a size of 1 mm in diameter [7]. In a clinical pilot study, we could localize occult insulinomas that were not detectable using conventional imaging methods [8]. In addition, the short-range Auger component of the compound has a strong therapeutic effect in animal models of human neuroendocrine cancer and resulted in a 94% reduction of the tumor mass after the injection of 28 MBq of [Lys^40^(Ahx-DTPA-^111^In)NH_2_]-exendin-4 in the Rip1Tag2 mouse model [6]. However, the injection of highly active [Lys^40^(Ahx-DTPA-^111^In)NH_2_]-exendin-4 in a dose of 28 MBq resulted in significant renal radiation damage and consequent chronic renal failure [6].

Thus, targeted cytotoxic and radioactive pharmaceuticals still have off-target effects on cells and tissues which do not express the receptor for the drug. Therefore, targeted therapy against neuroendocrine tumors may be more efficient and better tolerated if a cytotoxic targeted compound is combined with another targeted agent with a different toxicity profile.

Neuroendocrine tumors are well vascularized [9]. Our laboratory has previously shown that the expression of the vascular endothelial growth factor (VEGF) is upregulated in neuroendocrine tumors [10]. However, anti-angiogenic treatment as a monotherapy is not a promising option, since vascular regrowth and increased local tumor invasion after reversal of VEGF inhibition or through upregulation of fibroblast growth factors (FGF) are serious concerns [11-13]. Based on this, we instead hypothesize that the combination of targeted therapy against neuroendocrine tumor cells themselves in conjunction with anti-angiogenic compounds may increase the tumor response, reduce off-target effects, and prevent tumor resistance as encountered in monotherapy. As a second combination strategy, we also investigated the [Lys^40^(Ahx-DTPA-^111^In)NH_2_]-exendin-4 radiopeptide in combination with imatinib, a small molecule inhibitor of c-kit and PDGFR. Imatinib was shown to reduce the interstitial pressure inside tumor tissue and may thus increase the delivery of the radiopeptide to the tumor cells [14,15].

To test and compare these two drug combination strategies, we treated a mouse model of human neuroendocrine cancer, the Rip1Tag2 mouse, with low-dose (1.1 MBq) [Lys^40^(Ahx-DTPA-^111^In)NH_2_]-exendin-4 and either vatalanib (PTK787/ZK-222584) or imatinib (STI571) for 7 days. For adequate controls, all agents including the radiopeptide and the non-radioactive ^nat^In-exendin-4 were administered as monotherapy. Vatalanib is a potent inhibitor of all three members of the VEGFR family (VEGFR-1 to 3). Agents directed against VEGFR-2 have been shown to increase drug uptake in human tumor cell xenografts in athymic mice [16,17]. Vatalanib has proven effective in several animal models of human cancer [18]. However, vatalanib has also an effect on receptor tyrosine kinases (RTKs) outside the VEGFR family: it inhibits the platelet-derived growth factor receptor beta (PDGFR-β), c-kit, and the colony stimulating factor 1 receptor (CSF1R, also known as c-Fms), thus showing a certain overlap with imatinib.

The combination therapy was performed in the Rip1Tag2 mouse model of pNET. These mice express the simian virus (SV) large T antigen under the control of the rat insulin promoter (Rip) and develop β-cell tumors in a process of multi-stage carcinogenesis. Normal islets and pNETs express both the VEGFR-1 and VEGFR-2 selectively in CD31-positive endothelial cells [10]. Regardless of the tumor stage, the tumor cells themselves do not express significant levels of VEGFR-1 or VEGFR-2. PDGFR-β, the main target of imatinib in Rip1Tag2 mice, is expressed on pericytes covering the larger vessels in the tumor, but again not on the pancreatic tumor cells [19,20]. Notably, Rip1Tag2 mice have been instrumental in demonstrating the anti-tumor potential of a number of targeted compounds, including vatalanib and imatinib [21-24]. With this pre-clinical study, we provide evidence that the combination of a targeted radiopeptide with anti-angiogenic therapy provides a means of enhancing tumor response and reducing therapy toxicity in neuroendocrine tumors.

## Methods

### Reagents and instrumentation

All chemicals were obtained from commercial sources and used without further purification. ^111^InCl_3_ was purchased from Covidien Medical (Petten, Netherlands). Analytical HPLC was performed on a Metrohm HPLC system LC-CaDI 22-14 (Herisau, Switzerland), and a Berthold LB 509 flow-through γ-detector (Berthold Technologies, Bad Wildbad, Germany) with a Macherey-Nagel Nucleosil 120-3C-18 column (Düren, Germany). Matrix-assisted laser desorption/ionization mass spectrometry (MALDI-MS) measurements were done on a Voyager sSTR equipped with a Nd:YAG laser (355 nm; Applied Biosystems, Carlsbad, CA, USA). Quantitative γ-counting was performed on a COBRA 5003 γ-system well counter (Packard Instrument Company, Basel, Switzerland).

### Peptide synthesis and radiolabelling

[Lys^40^(Ahx-DTPA)NH_2_]-exendin-4 was custom-synthetized by Peptide Specialty Laboratories (Heidelberg, Germany). The peptide conjugate was characterized by MALDI-MS and RP-HPLC. An aliquot of 4 μg [Lys^40^(Ahx-DTPA)NH_2_]-exendin-4 was dissolved in sodium acetate buffer (0.4 M/pH 5.0), incubated with 100 MBq ^111^InCl_3_ at room temperature for 30 min, and then subjected to a quality control by analytical HPLC (eluents: A = 0.1% TFA in water and B = acetonitrile, flow 0.75 ml/min; 0 min 95% A, 30 min 55% A, 32 min 0% A, 34 min 0% A, 37 min 95% A).

### Mice

Phenotypic and genotypic analyses of Rip1Tag2 transgenic mice in a C57Bl/6 background have been described previously [25]. Rip1Tag2 and wild type C57Bl/6 J mice were used for organ toxicity studies. The animals were maintained and treated in compliance with the guidelines of the Swiss Veterinary Office and the Cantonal Veterinary office of Basel-Stadt (approval 2085 and 789).

### Biodistribution, therapy, and toxicity study in Rip1Tag2 mice

Male and female Rip1Tag2 mice, 19 to 32 g of weight and 11.6 ± 0.7 weeks old, were stratified according to their age and randomly assigned to the different cohorts. For biodistribution studies, the Rip1Tag2 mice were treated with oral vatalanib (dissolved in polyethylene glycol) or imatinib (dissolved in water) at a dose of 100 mg/kg daily for up to 7 days. Both vatalanib and imatinib were provided by Novartis (Basel, Switzerland). Days (0, 3, 5, and 7 days) after the beginning of vatalanib or imatinib treatment, the mice were injected through the tail vein with 185 kBq (10 ng peptide/2 pmol) [Lys^40^(Ahx-DTPA-^111^In)NH_2_]-exendin-4 (^111^In-DTPA-exendin-4) in a 100-μl 1% human serum albumin solution diluted in 0.9% NaCl. At 4 h after injection, the mice were sacrificed under isoflurane and CO_2_ anesthesia. The organs, blood, and tumors were collected, weighed, and the radioactivity in these samples was determined using a γ-counter. The percentage of injected activity (IA)/g tissue was calculated for each tissue.

To assess the efficacy of the combined therapy, Rip1Tag2 mice were injected once with 1.1 MBq (50 ng peptide/10 pmol) ^111^In-DTPA-exendin-4 i.v. just before the oral application of vatalanib or imatinib. Vatalanib and imatinib were then administered at a dose of 100 mg/kg daily for 7 days. On day 8, the mice were sacrificed, and tumor diameters were measured with a grid (for small tumors) or with a caliper (for large tumors). Afterward, tumor volumes were calculated, assuming a spherical shape of the tumors. Monotherapies with vatalanib, imatinib, 1.1 MBq ^111^In-DTPA-exendin-4, and 40 pmol non-radioactive [Lys^40^(Ahx-DTPA-^nat^In)NH_2_]-exendin-4 (^nat^In-exendin-4) were performed as controls. Kidney toxicity studies were carried out in Rip1Tag2 mice up to 30 days and in C57Bl/6 J mice up to 6 months after the termination of combination therapy and monotherapy. To this end, Rip1Tag2 and C57Bl/6 J were sacrificed, and the internal organs (pancreas, lungs, bowel, and kidneys) were removed and analyzed histologically (H&E and PAS). In addition, toluidine blue staining and electron microscopy of the kidneys were performed.

### Histological analysis

The kidneys and tumors were fixed in 4% paraformaldehyde overnight at 4°C and embedded in paraffin. Immunohistochemistry (including BrdU and TUNEL assays) on paraffin sections were performed as described previously [6]. For the staining of endothelial cells, rat anti-mouse CD31 (BD Pharmingen, Allschwil, Switzerland) was used.

### Autoradiography

GLP-1 receptor autoradiography was used to quantify the GLP-1 receptors in the mouse tumors in the presence or absence of vatalanib treatment. The Rip1Tag2 mice were treated with vatalanib as described before for 4 days and sacrificed on day 5. During necropsy, the tumors were snap frozen on dry ice. Tumor sections (20-μm thick) were incubated for 2 h at ambient temperature in the presence of 32 pM [^125^I]-GLP-1 (2,000 Ci/mmol). The incubation solution was 170 mM Tris-HCI buffer (pH 8.2) containing 1% bovine serum albumin, bacitracin (40 μg/ml) and MgCl_2_ (10 mM) to inhibit endogenous proteases. Non-specific binding was determined by adding 100-nM solution of unlabeled GLP-1. The incubated sections were washed twice for 5 min in cold incubation buffer containing 0.25% bovine serum albumin, then in buffer alone, and dried quickly. Finally, the sections were apposed to Biomax MR films (Kodak, Rochester, NY, USA) and exposed for 1 week in X-ray cassettes.

In all the experiments, the autoradiograms were quantified using a computer-assisted image processing system [26,27]. Tissue standards for iodinated compounds were used for this purpose.

### Radioligand internalization and nuclei isolation studies

A nuclei isolation kit (Nuclei EZ Prep Kit, Sigma-Aldrich Chemie Gmbh, Steinheim, Germany) was used to separate and quantify the amount of radioactivity stored in the nuclei [28]. The preparation was done according to the manufacturer's instructions. The GLP-1 receptor-expressing tumor cells were established from pancreatic tumors of Rip1Tag2 mice, as described previously [7]. The cells were seeded at a density of 2 × 10^7^ cells in Petri dishes and incubated overnight. Afterward, 25 pmol ^111^In-DTPA-exendin-4 (2.5 MBq) was added to the Petri dishes and incubated at 37°C. The internalization was stopped at 2, 4, and 24 h by removing the medium and by washing the cells four times with 5-ml ice-cold phosphate-buffered saline. Ice-cold nuclei EZ lysis buffer (2 ml) was added, and the cells were scraped with a small bladed cell scraper. The cell lysate was transferred to a separate 15-ml centrifuge tube and set on ice for 5 min. To collect the nuclei, the cell lysate was centrifuged three times at 500 g for 5 min at 4°C. The culture medium, the clear supernatant (cytoplasmatic supernatant), and nucleus pellets were measured radiometrically with a γ-counter.

### Fluorescence microscopy

Subcellular localization of exendin-4 in mouse pancreatic tumor cells was evaluated by fluorescence microscopy using fluorescein-Trp25-exendin-4 (ANAWA, Zurich, Switzerland) [29]. The GLP-1 receptor-expressing pancreatic tumor β-cells were freshly isolated from the Rip1Tag2 mice. They were cultured transiently and used within 6 weeks. Briefly, the cells were seeded into 12-well plates; one glass coverslip was added for each well and incubated overnight at 37°C. The day after, fluorescein-Trp25-exendin-4 (30 pmol, 100 μl) was added, and the plates were incubated for 2 h at 37°C. Immunofluorescence was performed as described previously [30]. We used a lysosome marker, the rat monoclonal antibody [GL2A7] against LAMP2 and an early endosome marker, the rabbit polyclonal antibody against Rab5. The plates were washed three times for 5 min per wash with HBS/Ca^2+^ and incubated for 30 min at room temperature (RT) in a dark room with the secondary antibodies (1:200 dilution in HBS/Ca^2+^). After washing, the fluorescent nuclear stain 4′, 6′-diamidino-2-phenylindole dihydrochloride (DAPI; Sigma-Aldrich, Saint Louis, MO, USA) was added, and the plates were incubated for 2 min at RT. Immunofluorescence microscopy was performed on a LSM 510 META confocal microscope (Zeiss, Oberkochen, Germany).

### Statistical analysis

Statistical analysis was performed using the GraphPad Prism software (GraphPad Software Inc., San Diego, CA, USA). The Kolmogorov-Smirnov test with Dallal-Wilkinson-Lillie for *p* value was used to assess the normality distribution of the data. Tumor volume and mass were calculated using non-parametrical statistical analysis (Kruskal-Wallis test) with Dunn's post-test. Proliferation, apoptosis, vessel density, and peptide uptake were analyzed by parametric testing (one-way ANOVA and Newman-Keuls post-test).

## Results

### Biodistribution of [Lys^40^(Ahx-DTPA-^111^In)NH_2_]-exendin-4

**Figure 1:**
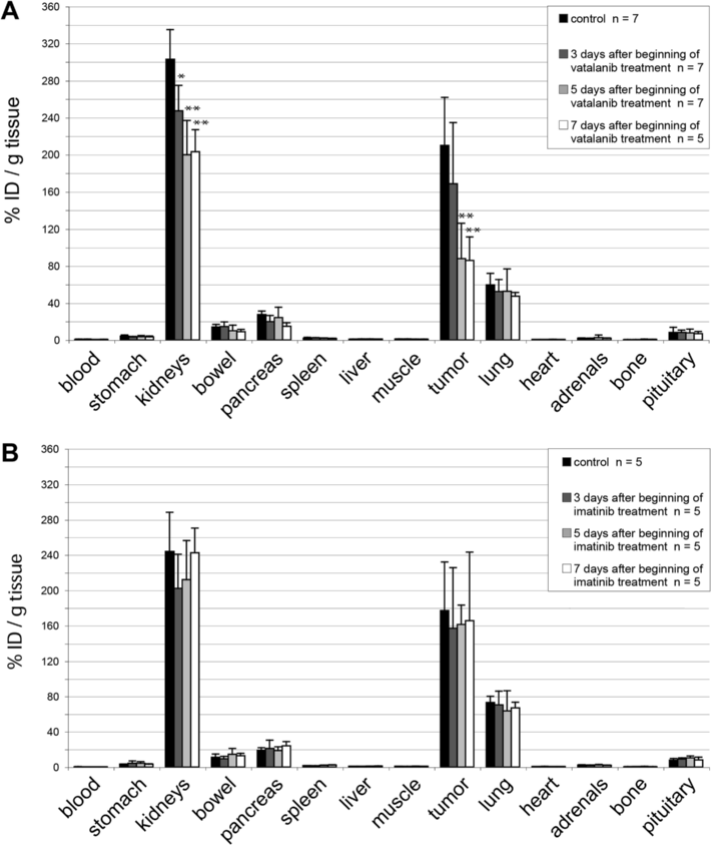
**Biodistribution of [Lys**^**40**^**(Ahx-DTPA-**^**111**^**In)NH**_**2**_**]-exendin-4. (A)** Treatment of Rip1Tag2 mice with the VEGFR2-inhibitor vatalanib induces a sharp decrease of [Lys^40^(Ahx-DTPA-^111^In)NH_2_]-exendin-4 uptake into the tumor tissue and kidneys. In particular, specific tumor uptake diminishes by more than 50% within the first 5 days of vatalanib treatment (**p < 0.01, *p < 0.05, Newman-Keuls post-test). **(B)** Pre-treatment of Rip1Tag2 mice with imatinib does not significantly affect the specific uptake of [Lys^40^(Ahx-DTPA-^111^In)NH_2_]-exendin-4 into the tumor tissue and organs.

We investigated the effect of a combination treatment with vatalanib and imatinib on the uptake of [Lys^40^(Ahx-DTPA-^111^In)NH_2_]-exendin-4 in the pancreatic tumors and other organs of Rip1Tag2 mice. Injection of vatalanib led to a significant decrease of intratumoral peptide uptake by 59.3% (86% IA/g versus 210% IA/g) within 7 days (*p* < 0.001, Newman-Keuls post-test, Figure 1A). After 3 days of vatalanib treatment, there was already a (statistically not significant) trend to lower radiopeptide uptake, and the uptake decreased further during the following 4 days. Interestingly, the uptake in the kidney was also reduced by 29.5% (*p* < 0.001, Newman-Keuls post-test), while the uptake in all the other organs was unaffected. In contrast, the pre-treatment of mice with imatinib did not affect the uptake of the radiopeptide, independent of the organ or time point analyzed (Figure 1B).

### Subcellular localization of [Lys^40^(Ahx-DTPA-^111^In)NH_2_]-exendin-4

**Figure 2:**
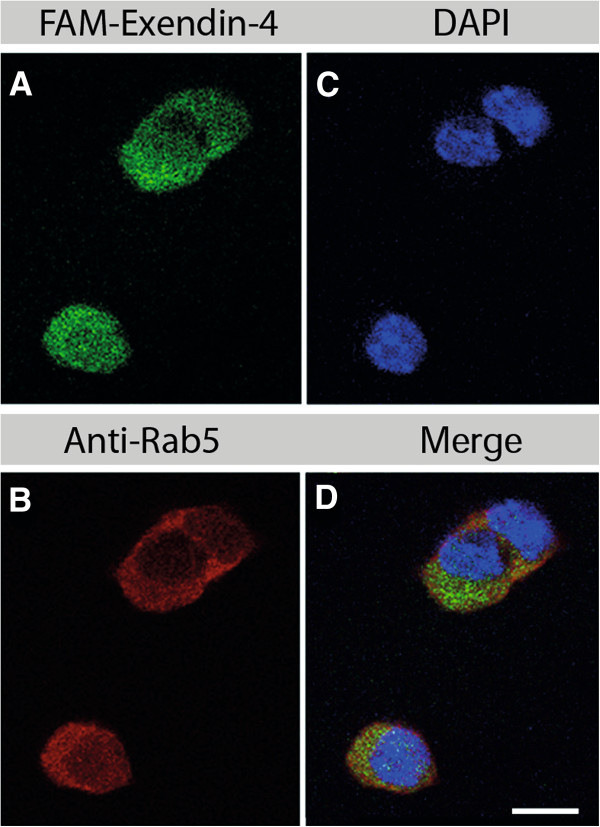
**Subcellular localization of fluorescein-Trp25-exendin-4 in cultivated tumor β-cells of Rip1Tag2 mice 2 h after injection.** Tumor beta-cells were incubated with fluorescein-Trp25-exendin-4 (**A**, green), anti-Rab5a (**B**, red, an anti-early endosome antibody), and nuclear DAPI stain (**C**, blue). Fluorescein-Trp25-exendin-4 was internalized into the cytoplasm and to a much lower degree into the nuclei of the tumor beta-cells. However, no strong co-localization was observed (**D**, merged picture). Scale bar, 10 μm.

**Figure 3:**
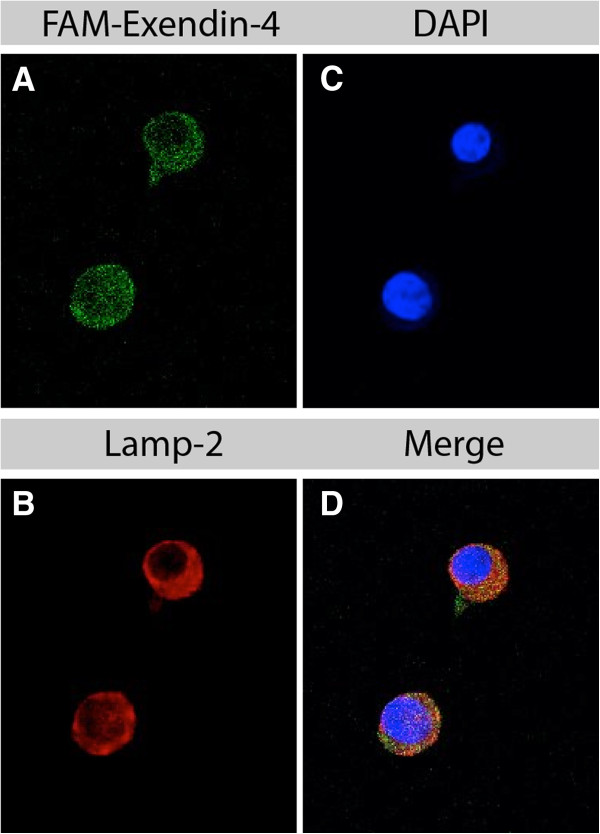
**Subcellular localization of exendin-4 in cultivated tumor β-cells of Rip1Tag2 mice 2 h after injection.** Tumor beta-cells were incubated with FAM-exendin-4 (**A**, green), anti-Lamp-2 (**B**, red, an anti-lysosome antibody), and nuclear DAPI stain (**C**, blue). Fluorescein-exendin-4 was internalized into the cytoplasm and into the nuclei of tumor beta-cells. However, there was only a minimal co-localization of FAM-exendin and Lamp-2 (**D**, merged picture).

The subcellular distribution was assessed to determine the internalization pathway and the amount of radioactivity stored in the nuclei after 2, 4, and 24 h of continuous incubation of pancreatic tumor cells with [Lys^40^(Ahx-DTPA-^111^In)NH_2_]-exendin-4. Immunofluorescence co-staining of the cultivated pancreatic tumor cells of Rip1Tag2 mice incubated 30, 60, 120, and 240 min with fluorescein-Trp25-exendin-4 and stained with antibodies against Rab5 (early endosome), Rab7 (late endosome), or LAMP-2 (lysosome) indicates that cellular uptake of exendin-4 is not strongly correlated with the endosomal (Figure 2) or the lysosomal pathway (Figure 3). At 4 h of incubation, the specific uptake into the cells was 9.78 ± 0.88% of the totally administered activity, increasing to 31.40 ± 5.04% at 24 h. Under these conditions, the nuclear uptake slightly increased over the time of incubation. After 2 and 4 h, only 1.0 ± 0.21% and 1.2 ± 0.3% of specifically internalized [Lys^40^(Ahx-DTPA-^111^In)NH_2_]-exendin-4 were localized in the cell nucleus, respectively. This increased to 3.0 ± 1.0% at 24 h (mean ± SD of three to six experiments).

### GLP-1R expression on β-cell tumors is not affected by vatalanib treatment

We next wanted to investigate a possible interaction between the treatment of the Rip1Tag2 mice with vatalanib and the expression of the GLP-1R. To this end, we pre-treated Rip1Tag2 mice for 4 days with either 250 μl of saline (control, *n* = 3) or with 100 mg/kg vatalanib p.o. (interventional group, *n* = 4), followed by an administration of [Lys^40^(Ahx-DTPA-^111^In)NH_2_]-exendin-4. Thereafter, the expression of the GLP-1R on pancreatic tumors was quantified using autoradiography. The expression of the GLP-1R was not influenced by the treatment with vatalanib (control 14,257 ± 7,070 dgm/min, vatalanib-treated 14,176 ± 5,958 dgm/min, *p* = 0.98, two-sided *t* test, data not shown). Thus, the reduced uptake of the radiopeptide, which is observed upon the treatment of mice with vatalanib, is not due to a direct effect of vatalanib on the expression of the GLP-1R but rather to a reduced delivery of the compound to the tumor cells.

### Therapeutic effects

**Figure 4:**
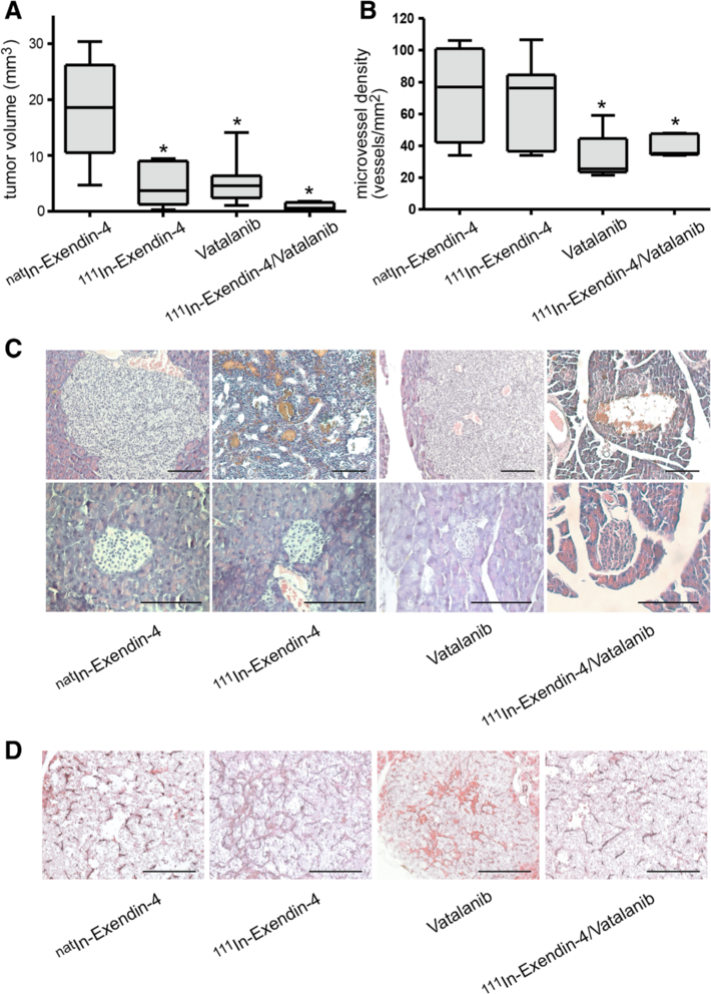
**Therapeutic efficiency of combination therapy with [Lys**^**40**^**(Ahx-DTPA-**^**111**^**In)NH**_**2**_**]-exendin-4 and vatalanib. (A)** The combination therapy reduces tumor burden by up to 97% (*p < 0.05, Kruskal-Wallis test) compared to the single-modality treatment. **(B)** The combination therapy and monotherapy with vatalanib reduce the microvessel density significantly compared to monotherapy with [Lys^40^(Ahx-DTPA-^111^In)NH_2_]-exendin-4 (*p* < 0.05, Newman-Keuls post-test). **(C)** H&E staining of tumors upon treatment. Histology confirms tumor regression in the combination therapy arm, while normal islets are unaffected. **(D)** CD31 immunohistochemical staining on tumor sections. Vessels persist under anti-angiogenic therapy, but vessel density is reduced. Size bars, 100 μm.

The combined treatment regimen consisting of 1.1 MBq [Lys^40^(Ahx-DTPA-^111^In)NH_2_]-exendin-4 and vatalanib led to a significant reduction of the tumor volume with a volume treated/volume control (T/C) ratio of 0.028 (2.8%) (Figure 4A, *p* < 0.0001 Kruskal-Wallis test). The combination was superior to the single-agent treatments with either vatalanib or 1.1 MBq [Lys^40^(Ahx-DTPA-^111^In)NH_2_]-exendin-4 alone (T/C 0.26 for vatalanib and T/C 0.21 for 1.1 MBq of the radiopeptide, *p* < 0.05, Dunn's test). The approximate 97% reduction of the tumor volume is similar to the effect observed after a single injection of 28 MBq [Lys^40^(Ahx-DTPA-^111^In)NH_2_]-exendin-4, i.e., at 25-times higher radioactive dosage [6]. The combined treatment modality led to the formation of hemorrhagic lacunae in the centers of the tumors without affecting morphologically normal islets (Figure 4C). The treatment of mice with vatalanib alone or vatalanib and the radiopeptide induced a significant decrease in microvessel density as assessed by quantification of CD31-positive blood vessels (median of 78/mm^2^ in the ^nat^In-exendin-4 group, 77/mm^2^ in the 1.1 MBq [Lys^40^(Ahx-DTPA-^111^In)NH_2_]-exendin-4 group, 28/mm^2^ for vatalanib, and 34/mm^2^ for the combination therapy; Figure 4B,D, *p* < 0.05, Newman-Keuls test).

**Figure 5:**
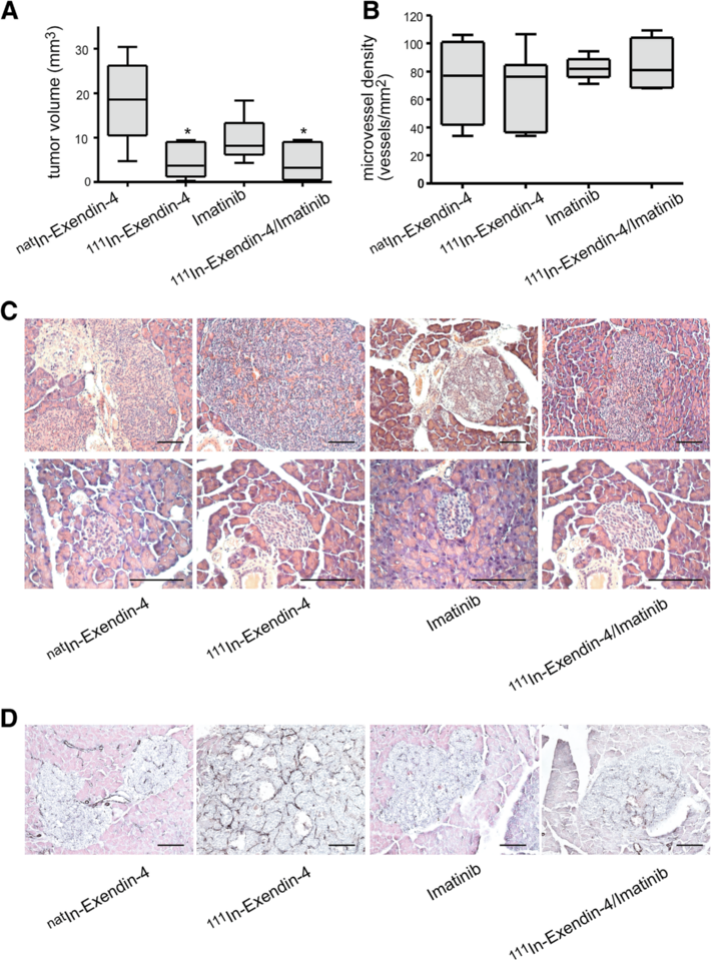
**Therapeutic efficiency of combination therapy with [Lys**^**40**^**(Ahx-DTPA-**^**111**^**In)NH**_**2**_**]-exendin-4 and imatinib. (A)** The combination of [Lys^40^(Ahx-DTPA-^111^In)NH_2_]-exendin-4 with imatinib has no additive effect on tumor burden (*p < 0.05, Kruskal-Wallis test). **(B)** The microvessel density is not affected by the (combination) therapy with imatinib. **(C)** H&E staining of tumor sections after the completion of therapy. Tumor necrosis can be seen under treatment with [Lys^40^(Ahx-DTPA-^111^In)NH_2_]-exendin-4. However, the combination treatment with imatinib does not alter the tumor morphology. **(D)** The microvessel density is unaffected by treatment with [Lys^40^(Ahx-DTPA-^111^In)NH_2_]-exendin-4 and/or imatinib. Size bars, 100 μm.

In contrast, the combination of 1.1 MBq [Lys^40^(Ahx-DTPA-^111^In)NH_2_]-exendin-4 and imatinib showed no therapeutic benefit (Figure 5). The radiopeptide alone was as efficient as the combination with imatinib (T/C for 1.1 MBq [Lys^40^(Ahx-DTPA-^111^In)NH_2_]-exendin-4 0.21, T/C for imatinib 0.42, and T/C for the combination 0.18; Figure 5A,C). Imatinib had no apparent effect on tumor-associated vessels, and the microvessel density was comparable in all four cohorts (median number of vessels between 78 and 81/mm^2^; Figure 5B,D).

### Effect of combination therapy on tumor cell proliferation and apoptosis

**Figure 6:**
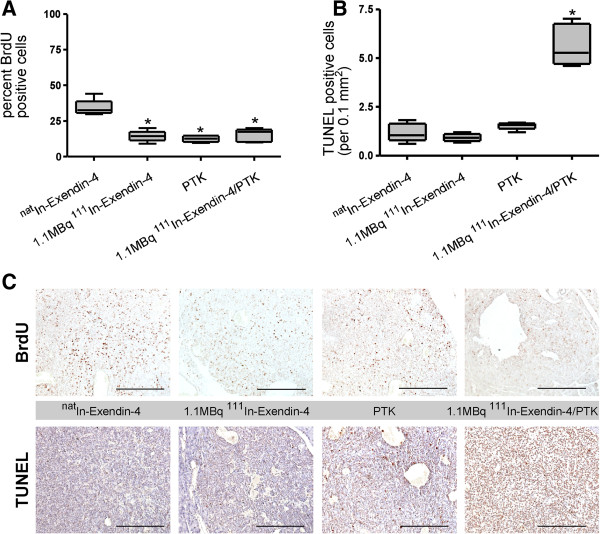
**Proliferation and apoptosis of tumor β-cells treated with [Lys**^**40**^**(Ahx-DTPA-**^**111**^**In)NH**_**2**_**]-exendin-4 and/or vatalanib. (A)** Treatment of mice with 1.1 MBq [Lys^40^(Ahx-DTPA-^111^In)NH_2_]-exendin-4, vatalanib, and the combination therapy induces a significant decrease in tumor cell proliferation (***p < 0.001, Newman-Keuls post-test). However, there is no significant difference between the three interventional groups. **(B)** In contrast, tumor cell apoptosis is significantly increased in the combined treatment group as compared to all the other groups (***p < 0.001, Newman-Keuls post-test). **(C)** Immunohistochemical staining for BrdU (proliferation, top row) and TUNEL (apoptosis, bottom row). Size bars, 100 μm.

**Figure 7:**
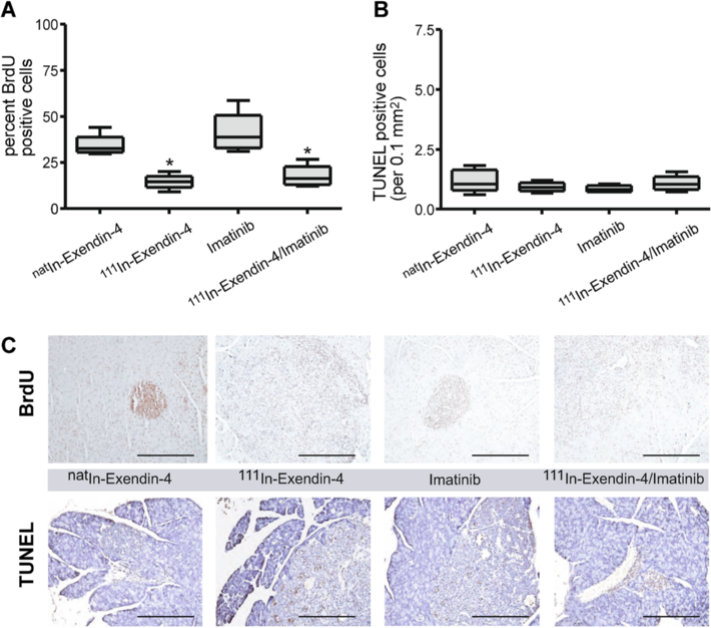
**Proliferation and apoptosis of tumor β-cells treated with [Lys40(Ahx-DTPA-111In)NH2]-exendin-4 and/or imatinib. (A)** Proliferation of tumor beta-cells of Rip1Tag2 mice treated with [Lys40(Ahx-DTPA-111In)NH2]-exendin-4 and [Lys40(Ahx-DTPA-111In)NH2]-exendin-4 plus imatinib is reduced compared to the ^nat^In-exendin-4 and the imatinib group (*p < 0.05, Newman-Keuls post-test). **(B)** However, the combined treatment modality does not increase tumor cell apoptosis compared to the other groups (*p* > 0.05, Newman-Keuls post-test). **(C)** Immunohistochemistry for proliferation (BrdU, top row) and apoptosis (TUNEL, bottom row) on the sections of Rip1Tag2 pancreas treated as indicated on the graph. Size bars, 100 μm.

To investigate the mechanism underlying the tumor response, we quantified tumor cell proliferation and apoptosis in all cohorts (Figures 6 and 7). The combination of vatalanib and 1.1 MBq [Lys^40^(Ahx-DTPA-^111^In)NH_2_]-exendin-4 did not significantly influence the rate of proliferating cells as compared with the single-agent treatments (31% of BrdU positive cells in the ^nat^In-exendin-4 group as compared to a median of 12% to 17% in all other groups; Figure 6A,C). In contrast, the number of apoptotic tumor cells in the combination group was increased fourfold in comparison to the cohorts injected with ^nat^In or 1.1 MBq [Lys^40^(Ahx-DTPA-^111^In)NH_2_]-exendin-4 alone, and threefold in face of the vatalanib regimen (median number of apoptotic cells/0.1 mm^2^ 1.3 in the ^nat^In-exendin-4 group, 1.2 in the 1.1 MBq [Lys^40^(Ahx-DTPA-^111^In)NH_2_]-exendin-4 cohort, 1.7 in the vatalanib group, and 5 in the combination therapy cohort; Figure 6B,C; *p* < 0.0001 one-way ANOVA; *p* < 0.001 Newman-Keuls test for all comparisons to the combined vatalanib + radiopeptide group).

The combination of imatinib with [Lys^40^(Ahx-DTPA-^111^In)NH_2_]-exendin-4 did not induce a significant change in the rate of proliferating or apoptotic cells as compared to the injection of 1.1 MBq [Lys^40^(Ahx-DTPA-^111^In)NH_2_]-exendin-4 alone (*p* > 0.05, Newman-Keuls test; Figure 7A,B,C).

Taken together, the combination of vatalanib with [Lys^40^(Ahx-DTPA-^111^In)NH_2_]-exendin-4 caused a sustained induction of apoptosis that was superior to single-agent treatment, exceeding a merely additive effect, while proliferation is not significantly affected in comparison to single-agent application. Adding imatinib to the radiopeptide had no effect on either parameter.

### Toxicity

To evaluate the toxicity of the combination treatments, we injected both Rip1Tag2 and C57/Bl6 mice with [Lys^40^(Ahx-DTPA-^111^In)NH_2_]-exendin-4 and treated both groups with either 100 mg/kg vatalanib or 100 mg/kg imatinib orally for 7 days. The Rip1Tag2 mice were sacrificed after 8 and 30 days, while the C57/Bl6 mice were analyzed after 8, 30, and 60 days and 6 months. Since the transgenic expression of the SV40 large T antigen continues in β-cells of treated Rip1Tag2 mice, these mice form new tumors after the end of the treatment, thus precluding long-term toxicity analysis in this model. Instead, long-term toxicity was assessed in the C57/Bl6 mice.

As shown in Figure 1, significant uptake of the radiopeptide is present in the pancreas, lungs, bowel, and kidneys. Furthermore, we had shown previously that the kidneys are the dose-limiting organs when treating pancreatic tumors in Rip1Tag2 mice [6]. We therefore analyzed the toxicity at the given time points through standard histology of the pancreas, lungs, bowel, and kidneys. No toxicity could be observed. After toluidine blue staining and electron microscopy of the kidneys, again, we found no indication of acute or chronic radiation damage or any other kidney pathology as assessed by histology.

## Discussion

Vascular normalization is defined as a remodeling of tumor-associated vessels into mature vessels, resulting in a more physiological morphology and function. Normalization is a well-established phenomenon, which occurs upon the treatment of malignant tumors with anti-angiogenic compounds and results in an improved oxygenation of the tumor, reduced vessel leakiness, and a normalized interstitial pressure [16,31]. This was shown to increase the tumor's response to either radiation therapy or cytostatic treatment. In a clinical setting, however, the question is not only if normalization occurs but also if the duration of the normalization lasts long enough to provide a therapeutic window. Clinical trials investigating the ‘window of opportunity’ are generally missing. Our data derived from biodistribution experiments in the Rip1Tag2 mouse indicate that at least in the presented experimental model, there is no such window. In our animal model, vatalanib reduced the drug uptake into the tumor cells, which may impair the efficacy of any targeted therapies. The reduced tumor uptake can be explained by the significant decrease of the microvessel density after treatment with vatalanib. This is in line with previous data, indicating that anti-angiogenic treatments indeed significantly reduce the uptake of targeted therapies in patients and in several animal models [32-36].

Interestingly, in a phase-three trial of sunitinib, there was an improvement of the overall and the progression-free survival in patients with pNET [37]. Like vatalanib, sunitinib is a multi-targeted tyrosine kinase inhibitor blocking all PDGF-Rs and VEGFRs. Thus, combination of targeted radiopeptides with anti-angiogenic compounds is a particularly attractive option because radiopeptide monotherapies such as [^90^Y-DOTA-Tyr^3^]-octreotide and [^177^Lu-DOTA-Tyr^3^]-octreotate showed an excellent objective response rate and progression-free survival in patients with neuroendocrine tumors [3,4]. In addition, cediranib, a highly potent inhibitor of VEGFR tyrosine kinase activity, showed a radiosensitizing effect in combination with radiotherapy [38]. In contrast, [^177^Lu-DOTA-Tyr^3^]-octreotate was shown to be less effective when combined with the mTOR inhibitor everolimus (RAD001, Novartis) in a rat model of pancreatic cancer [39]. Along the same line, no synergistic effect of adding imatinib was observed in our pre-clinical trial. This underlines the importance to choose adequate combination partners for radiopeptide therapy. Since both vatalanib and imatinib inhibit PDGFR, this argues that the main synergistic effect of the combination therapy is mediated via the inhibition of the VEGF receptors. For a potential clinical combination therapy, VEGFR inhibitors may thus be more suitable partners for radiopeptides than other kinase inhibitors.

Unfortunately, the value of the Rip1Tag2 mice as a long-term treatment model is rather limited. Because of the ongoing expression of the large T antigen in β-cell islets, these mice continue to form new tumors during their whole lifespan [25]. In a previous set of experiments, our group had shown that monotherapy with vatalanib has a persistent effect on the vessel formation for up to 21 days in the Rip1Tag2 model. However, there is no further decrease of microvessel density when the treatment is prolonged [40]. The reduction of the tumor volume was similar to that for a 1-week and a 3-week course of therapy. Therefore, we have not performed prolonged treatment in this study.

This study has shown that the combination of GLP1-R targeted Auger emitter radiation therapy with an anti-angiogenic compound has a synergistic effect on the pancreatic tumors of the Rip1Tag2 transgenic mice. Of the many treatment regimens tested in the Rip1Tag2 mice, including genetic ablation or sequestration of tumor- and angiogenesis-promoting factors (VEGF, insulin-like growth factor II, and fibroblast growth factors), and classical chemotherapy, the combined treatment with [Lys^40^(Ahx-DTPA-^111^In)NH_2_]-exendin-4 and vatalanib is one of the most efficient, with up to 97% smaller tumor volume in the interventional group, compared to 67% to 91% in previous studies [6]. These results are comparable to those achieved using a 25-fold higher dose of [Lys^40^(Ahx-DTPA-^111^In)NH_2_]-exendin-4 in the absence of an anti-angiogenic compound [6].

Our results indicate that Auger radiation in combination with the inhibition of VEGF receptors is likely responsible for the massive induction of tumor cell apoptosis. Importantly, this effect is tumor cell specific since an extensive toxicological evaluation did not provide any evidence of acute or chronic organ damage with the combination treatment. Thus, the combination with an anti-angiogenic drug that has a different toxicity profile than the radiopeptide reduces the amount of [Lys^40^(Ahx-DTPA-^111^In)NH_2_]-exendin-4 and is a more nephroprotective treatment. This is relevant because kidney toxicity is a main limitation of targeted radiopeptide therapy [4,41]. Collectively, our data support the contention that VEGFR inhibition can enhance radiation response in tumors [38]. Although the mechanisms underlying the radiosensitizing effect of vatalanib remain unclear, it is conceivable that enhanced apoptosis together with reduced proliferation of tumor cells may reduce intratumoral oxygen demand to a level that can still be met by the damaged tumor vasculature and thereby avoid a hypoxic state in the tumor that would reduce the efficacy of [Lys^40^(Ahx-DTPA-^111^In)NH_2_]-exendin-4. Auger electron emitters such as ^111^In are known to damage the DNA if they decay close to the cell nucleus, and the potential advantage in targeted radionuclide therapy is its short range of nanometers to micrometers which is sufficient for damaging the DNA in tumor cells while sparing surrounding normal cells [42]. Goddu et al. estimates that the radiation dose to the nucleus in a single cell is increased twofold if ^111^In is located in the cytoplasm compared to the cell surface and about 20-fold if located in the nucleus [43]. Cai et al. used a three-dimensional cell cluster model, which is a more appropriate model for the microdose estimation of tumors *in vivo*. They showed that the estimated radiation dose to the nucleus is increased only twofold if ^111^In is located in the nucleus compared to the cytoplasm and cell surface [44]. In our experimental model, less than 5% of the internalized activity accumulated in the nucleus. Therefore, the therapeutic effect of 1.1 MBq [Lys^40^(Ahx-DTPA-^111^In)NH_2_]-exendin-4 can be explained by the very high tumor uptake of 287 ± 62% IA/g tumor [7]. A rough calculation allows the estimation that approximately 9 mBq is taken up by a single tumor cell (4,460 kBq/g tumor, 1-gram wet weight is assumed to contain 5 × 10^8^ tumor cells). This value appears low, but is higher than what Chen et al. found using an ^111^In-NLS-HuM195 antibody targeting the CD33 antigen overexpressed on acute myeloid leukemia cells [45]. In cell culture, they found a major reduction of cell survival with 1.48 mBq ^111^In/cell if about 50% of ^111^In-NLS-HuM195 is located in the nucleus.

## Conclusions

In our study, we found that the combination of 1.1 MBq of [Lys^40^(Ahx-DTPA-^111^In)NH_2_]-exendin-4 with 100 mg/kg oral vatalanib for 7 days had the same effect on a neuroendocrine tumor as the single-injection of 28 MBq of the radiopeptide alone. Our data suggest that this co-treatment is synergetic and at the same time reduces side effects, such as radiation damage of the kidneys. Many neuroendocrine tumors have a high density of tumor-associated vessels, and thus our model might serve as paradigm, highlighting the potential of a combined treatment against neuroendocrine tumor vessels and tumor cells alike. In contrast, the addition of imatinib did not result in any additive or synergistic therapeutic effect.

Based on these results, we deem it warranted to evaluate this approach in a clinical trial. A clinical confirmation of these data will hopefully help advance a more efficient therapy of neuroendocrine tumors with less off-target effects in the future.

## Competing interests

The authors declare that they have no competing interests.

## Authors’ contributions

AW and DW proposed the study and participated in drafting the experiments, performing the experiments, and writing the article. VP participated in reviewing the data, producing the figures, and writing the article. RM performed the intracellular localization experiments. AO participated in producing the figures and writing the article. JR identified the GLP1 receptor and participated in drafting the experiments and analyzing the data. CR participated in drafting the *in vivo* experiments, analyzing the data, and writing the article. CM participated in drafting and performing the experiments and writing the article. HM produced the GLP1-binding radiopeptide and participated in drafting the experiments and writing the article. GC participated in drafting the *in vivo* experiments, analyzing the data, and writing the article. All authors read and approved the final manuscript.

## Authors’ information

AW, CR, and CM are medical oncologists. DW has specialized in nuclear medicine. RM and HRM are radiochemists. VP, AO, and GC are molecular biologists. JCR is a pathologist.
